# Potential Role of Biofilm Formation in the Development of Digestive Tract Cancer With Special Reference to *Helicobacter pylori* Infection

**DOI:** 10.3389/fmicb.2019.00846

**Published:** 2019-04-29

**Authors:** Cosmeri Rizzato, Javier Torres, Elena Kasamatsu, Margarita Camorlinga-Ponce, Maria Mercedes Bravo, Federico Canzian, Ikuko Kato

**Affiliations:** ^1^Department of Translation Research and of New Technologies in Medicine and Surgery, University of Pisa, Pisa, Italy; ^2^Unidad de Investigación en Enfermedades Infecciosas, Unidades Médicas de Alta Especialidad Pediatría, Instituto Mexicano del Seguro Social, Mexico City, Mexico; ^3^Instituto de Investigaciones en Ciencias de la Salud, National University of Asunción, Asunción, Paraguay; ^4^Grupo de Investigación en Biología del Cáncer, Instituto Nacional de Cancerología, Bogotá, Colombia; ^5^Genomic Epidemiology Group, German Cancer Research Center (DKFZ), Heidelberg, Germany; ^6^Department of Oncology and Pathology, Wayne State University School of Medicine, Detroit, MI, United States

**Keywords:** *Helicobacter pylori*, biofilm, persistent infection, cancer, virulence

## Abstract

Bacteria are highly social organisms that communicate via signaling molecules and can assume a multicellular lifestyle to build biofilm communities. Until recently, complications from biofilm-associated infection have been primarily ascribed to increased bacterial resistance to antibiotics and host immune evasion, leading to persistent infection. In this theory and hypothesis article we present a relatively new argument that biofilm formation has potential etiological role in the development of digestive tract cancer. First, we summarize recent new findings suggesting the potential link between bacterial biofilm and various types of cancer to build the foundation of our hypothesis. To date, evidence has been particularly convincing for colorectal cancer and its precursor, i.e., polyps, pointing to several key individual bacterial species, such as *Bacteroides fragilis, Fusobacterium nucleatum*, and *Streptococcus gallolyticus* subsp. *Gallolyticus.* Then, we further extend this hypothesis to one of the most common bacterial infection in humans*, Helicobacter pylori* (*Hp*), which is considered a major cause of gastric cancer. Thus far, there has been no direct evidence linking *in vivo Hp* gastric biofilm formation to gastric carcinogenesis. Yet, we synthesize the information to support an argument that biofilm associated-*Hp* is potentially more carcinogenic, summarizing biological characteristics of biofilm-associated bacteria. We also discuss mechanistic pathways as to how *Hp* or other biofilm-associated bacteria control biofilm formation and highlight recent findings on *Hp* genes that influence biofilm formation, which may lead to strain variability in biofilm formation. This knowledge may open a possibility of developing targeted intervention. We conclude, however, that this field is still in its infancy. To test the hypothesis rigorously and to link it ultimately to gastric pathologies (e.g., premalignant lesions and cancer), studies are needed to learn more about *Hp* biofilms, such as compositions and biological properties of extracellular polymeric substance (EPS), presence of non-*Hp* microbiome and geographical distribution of biofilms in relation to gastric gland types and structures. Identification of specific *Hp* strains with enhanced biofilm formation would be helpful not only for screening patients at high risk for sequelae from *Hp* infection, but also for development of new antibiotics to avoid resistance, regardless of its association with gastric cancer.

## Introduction

Over the past two decades, it has been increasingly appreciated that bacteria present in most biological systems exist in biofilms, which are defined as matrix-enclosed microbial accretions adhering to biological or non-biological surfaces and to each other (Hall-Stoodley et al., 2004; Gabrilska and Rumbaugh, 2015; Flemming et al., 2016). Biofilm formation is a key factor for survival in diverse environments and is viewed as an ancient and integral component of the prokaryotic life cycle, as researchers found biofilm formation early in fossil records (∼3.25 billion years ago) (Hall-Stoodley et al., 2004). In biofilms, unicellular bacteria assume a temporary multicellular lifestyle through prolific intercellular interactions, both social and physical, immersing in a complex and specialized matrix formed by both the bacteria and the host (Kostakioti et al., 2013; Watters et al., 2016). These group behaviors represent a microbial social support system to foster survival in hostile environments, share limited resources, favor long-term persistent colonization and maintain the ability to colonize to new niches (Hall-Stoodley et al., 2004; Majumdar and Pal, 2017). Accordingly, this specific mode of living provides strong fitness advantage to biofilm-associated bacteria compared to their planktonic counterparts (Gabrilska and Rumbaugh, 2015; Liu et al., 2016). In particular, biofilm-associated bacteria exhibit increased resistance to chemical disinfectants, antibiotic therapies and human immune responses and thus are associated with long-term persistence (Elias and Banin, 2012; Kostakioti et al., 2013). The self-produced extracellular polymeric substance (EPS) matrix, which is typically composed of polysaccharides, carbohydrate-binding proteins, lipids, extracellular DNA (eDNA), pili, flagella, and other adhesive fibers (Kostakioti et al., 2013; Flemming et al., 2016), plays an important role in antibiotic resistance, enhanced horizontal gene transfer as well as altered gene expressions within the biofilm community, which results in enhanced bacterial virulence (Elias and Banin, 2012; Madsen et al., 2012; Flemming et al., 2016). Furthermore, it has been described that some extracellular pathogens adopt intracellular lifestyle through the formation of bacterial communities with biofilm-like properties, enabling them to persist inside the host cells (Kostakioti et al., 2013). Thus, these biofilm-associated infections in humans on both abiotic (medical devices) and biotic surfaces (e.g., gum, heart valves, lungs, etc.) pose significant challenges to the medical community (Costerton et al., 1999; Hall-Stoodley et al., 2004; Kostakioti et al., 2013).

Chronic inflammation, often caused by chronic microbial infection, has been decisively linked to several stages of carcinogenesis (Grivennikov et al., 2010; Armstrong et al., 2018). A recent estimate also suggests that about 15% of worldwide incident cases of cancer are attributable to chronic infection (Plummer et al., 2016). As discussed above, one way for bacteria to achieve persistent colonization in the hosts is to form biofilms. Despite well-established causal links between certain infectious agents, such as *Helicobacter pylori* (*Hp*), and cancer (Plummer et al., 2016), knowledge concerning the association between bacterial biofilm formation and cancer development has been sparse.

Despite the well-founded association between persistent *Hp* infection and gastric carcinogenesis and growing knowledge concerning unique properties of biofilm-associated bacteria, little is known about biological consequence of *Hp in vivo* biofilm formation beyond antibiotic resistance. In the subsequent sections, we present our hypothesis that biofilm formation has potential etiological role in the development of digestive tract cancer, which may be particularly relevant to infection-associated cancer, such as *Hp*-induced gastric cancer. We synthesize recent new findings suggesting the potential link between bacterial biofilm and various types of cancer, as well as the information to support an argument that biofilm associated-*Hp* is potentially more carcinogenic. We also discuss technical details and challenges in *Hp* biofilm studies to aid interpretation of the results from various different experimental platforms and designing future studies and highlight recent findings on *Hp* genes and virulence factors that influence biofilm formation.

## Evidence to Support Possible Links Between Biofilm Formation and Human Cancer

Recently, a group from Johns Hopkins University in the US published a series of studies addressing the potential link between biofilm-associated bacteria and colorectal cancer and its precursor, i.e., polyps. The investigators found more frequent polymicrobial biofilm formation in colorectal mucosa of the patients with colorectal cancer or adenoma, compared with that of control subjects who had negative findings at screening colonoscopy. This phenomenon was striking for the right sided tumors compared to left sided lesions (Dejea et al., 2014; Drewes et al., 2017). Importantly, biofilms were present not only on tumors but also at normal surgical margins. Furthermore, biofilm formation was associated with diminished epithelial cell E-cadherin, enhanced IL-6 and Stat3 activation, and increased crypt cell proliferation in normal mucosa (Dejea et al., 2014). The subsequent analysis revealed that these tumor-associated biofilms were enriched with *Bacteroides fragilis (Bf)* and several periodontal pathogens, including *Fusobacterium nucleatum (Fn)* and *Peptostreptococcus stomatits*, and accompanied with altered functions, compared to those in planktonic bacteria, e.g., increases in cytoskeletal proteins, peptidoglycan (PG) biosynthesis and sporulation, and a decrease in flagellar assembly (Drewes et al., 2017). Biofilms were detected also in colorectal mucosa of genetically predisposed individuals, i.e., familial polyposis coli patients, regardless of the locations at the colorectum, but their appearance and composition were different (Dejea et al., 2018). They were rather patchy (as opposed to continuous as observed for sporadic cases) and primarily composed of *Bf* and polyketide-peptide genotoxin producing *pks* island positive *Escherichia coli*, with little periodontal pathogens (Dejea et al., 2018). It was also reported previously that *Bf* was the main component of inflammatory bowel disease-associated biofilms (Swidsinski et al., 2005).

Another piece of corroborative information arises from growing recognition of the association between oral microbiome, specifically *Fn*, and colorectal cancer (Sun and Kato, 2016), which was predominantly observed for proximal colon tumors (Hussan et al., 2017). *Fn* is a normal residential member of dental plaques (biofilms) and well-known periodontal pathogen (Larsen and Fiehn, 2017). *Fn* is considered to be a co-aggregation expert, with an ability of co-aggregating with a broad range of bacteria, nearly all bacterial species involved in oral plaque formation (Kolenbrander et al., 1989; Allen-Vercoe et al., 2011), a very important property in biofilm formation. Moreover, *Fn* can bind to and transport otherwise non-invasive bacterial species into host cells, acting as a shuttle in this respect (Edwards et al., 2006). *Fn* produces outer membrane vesicles (OMV) to facilitate coaggregation and isolated OMVs alone have been shown to exert an equivalent ability to coaggregate other bacteria compared to whole bacterial culture (Kinder and Holt, 1993). OMV production appears to depend on external stimuli and resulting changes in biofilm formation are strain-specific (Musrati et al., 2016). In this context, the putative association with colorectal cancer may be a function of the microbes that *Fn* gathers in its biofilms, rather than a direct effect of its own virulence. In fact, Flemer et al. (2018) more recently reported that several microbes commonly found in oral biofilms were enriched in colonic mucosa from colorectal cancer patients. Finally, *Fn* has recently found to be one of the gut microbes linked to pancreatic cancer (del Castillo et al., 2019), although little has been known about biofilm formation in the pancreatic ducts.

Moreover, growing evidence suggest a link between colorectal cancer and *Streptococcus gallolyticus* subsp. *Gallolyticus (SGG)*, formerly known as *S. bovis*, an opportunistic pathogen causing biofilm-associated infections, e.g. infective endocarditis on cardiac valves (Boleij et al., 2010, 2012; Sun and Kato, 2016; Butt et al., 2018; Jans and Boleij, 2018). Martins el al. have revealed that this bacterium exploits Pil3 pilus for adhesion to colonic mucus and for colonization of mouse distal colon (Danne et al., 2011; Martins et al., 2016), while Pil1 pilus allows *S. gallolyticus* to bind to collagen type I and plays a role in biofilm formation (Danne et al., 2011; Martins et al., 2016). Pil3 pilus has been shown to bind not only to human colonic mucins and to human stomach mucins, but also to human fibrinogen (Martins et al., 2016). Accordingly, both pilus proteins play an important role in biofilm formation. Binding to fibrinogen is also known to contribute to increased biofilm formation in other *Streptococcus* species (Bedran et al., 2013). Comparative genomics of 8 *SGG* strains form human blood and feces recently revealed that complete *pil1-3* loci are only present in virulent strains, causing bacteremia and/or endocarditis translocated through impaired mucosal barrier, while other fecal *SGG*s do not carry these full *pil1-3* loci (Jans and Boleij, 2018). Interestingly, several epidemiological studies, including our own, have found that individuals who express high antibody titers to several *S. gallolyticus* pilus proteins have increased risk of colorectal cancer (Boleij et al., 2012; Butt et al., 2016; Butt et al., 2018).

Gallbladder is another potential niche where bacteria can form biofilm. In fact, certain strains of *Salmonella typhi* are known to produce biofilms on the surfaces of cholesterol gallstone (Crawford et al., 2010; Gonzalez-Escobedo and Gunn, 2013), which is in support of the known etiological link between the carriages of *S. typhi* and gallstones and the development of gallbladder cancer (Di Domenico et al., 2017).

Gastric lumen is one of the most hostile environments in human body, which kills many bacteria within a few minutes. *Hp* survives in this environment with its urease activity that neutralizes gastric acid (Abadi, 2017), whereas biofilm formation may be more vital for its persistent colonization. Surprisingly, to date, little has been known about *in vivo* biofilm formation on human gastric mucosa. Three pioneer studies employed high powered electron microscope and demonstrated dense clusters of *Hp* (Carron et al., 2006; Coticchia et al., 2006; Cellini et al., 2008), primarily in coccoid forms, which are known to be viable but non-cultivable (Cellini, 2014; Percival and Suleman, 2014). It is interesting to note that *Hp* isolated from gastric cancer patients is often non-cultivable, despite the fact that the bacteria are detectable by other methods (i.e., PCR or histology), and that coccoid forms have been indeed more frequently found in gastric mucosa of gastric cancer patients than in that of peptic ulcer patients (Chan et al., 1994).

## Evidence to Support *Hp* Ability in *In Vitro* and *In Vivo* Biofilm Formation

### *In vitro* Abiotic Models

In 1999, Stark et al. (1999) reported the formation of biofilm by *Hp* in a continuous culture of *Hp* NCTC 11637 in Brucella broth supplemented with B-cyclodextrin and glucose in a glass fermentor. *Hp* formed a biofilm containing polysaccharides at the air-liquid interface. Later, Cole et al. (2004) and Bellack et al. (2006) described the progression of biofilm formation with clinical isolates of *Hp* showing that all strains tested were able to form biofilm at the air-liquid interface on a glass surface, although *Hp* biofilms have also been detected by molecular methods on the surface of environmental water supply system. Further studies have clarified that biofilms are not simply passive aggregates of cells attached to a surface, but they do complex communitarian functions and should be considered as biological systems.

The ability of *in vitro* biofilm formation by *Hp* has been described in several systems. These studies have demonstrated important differences in the *in vitro* growth conditions of the bacteria and in the abiotic surfaces used in the assay. Microtiter polystyrene plates are the most commonly-used substrate (Azeredo et al., 2017). The biomass attached to the surface of the wells and the formed biofilm is quantitated by staining with violet crystal (Wilson et al., 2017). Yonezawa et al. (2009) observed biofilm formation at the air-liquid interface of microtiter plates. More specific pieces of information regarding the structure of *Hp* biofilms *in vitro* on abiotic surface have also been made available by use of scanning electron microscopy (SEM) (Queralt and Araujo, 2007; Yonezawa et al., 2009, 2013). These include *Hp* morphological changes associated with biofilm formation in an aquatic culture model as well as how culture conditions and nutrient supply affect biofilm density, structures and abundance of OMVs. It is worth noting that OMVs have been found to enhance oxidative stress and genomic damages to host cells and thus possess carcinogenic potential in *in vitro* culture systems (Chitcholtan et al., 2008). Using transmission electron microscope analysis (TEM), Grande et al. demonstrated the presence of eDNA, which was associated with OMVs and plays a role in Hp aggregation (Grande et al., 2015).

**FIGURE 1:**
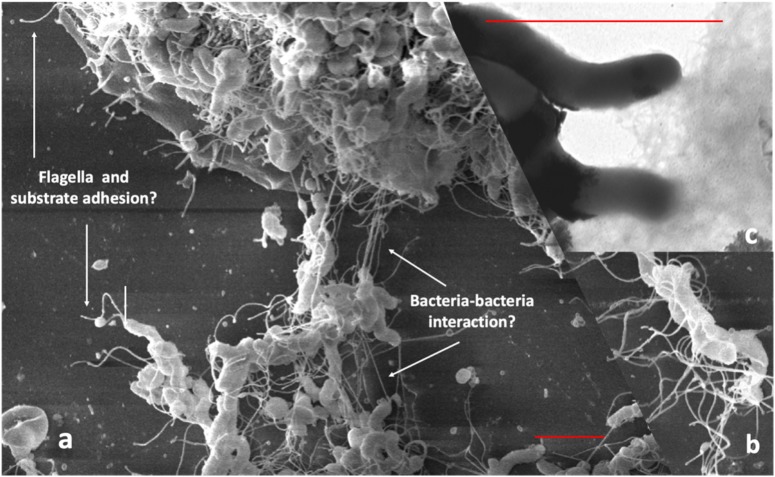
Scanning electron microscopy of *Hp* strain ATCC 43504 biofilm on a glass substrate. A bacterial suspension of 3 × 10^8^ CFU/ml in Brucella broth was applied to a glass slide in a 24-well plate, incubated, dried, fixed with methanol and processed for SEM, **(a)** Images showing flagella probably adhering to the substrate; **(b)** possible bacteria-bacteria interaction with the flagella; **(c)** pilli produced by *H. pylori* during biofilm formation. Red bars in **(a,c)** indicate approximately three-micrometer scale.

To demonstrate the interaction of bacteria and the role of cellular appendices in the formation of biofilm using SEM, we cultured *Hp* on glass cover slips for 48 h in RPMI medium (JT, MC, and J Girón, unpublished results). We observed a dense growth of bacteria attached to the substrate (Figure 1A), with an extensive expression of flagella. The images suggest that flagella might have a role in both, adhesion to the substrate and in the interaction between bacteria (Figure 1A,B). Images also show a high formation of smaller pili by bacteria forming the biofilm (Figure 1C). The role of flagella and other pili in the formation of biofilm has been well documented in other bacteria (Belas, 2014; Maldarelli et al., 2016; Zeng et al., 2017).

### *In vitro* Biotic Models

*Hp* forms biofilms not only on abiotic surfaces, but also on biotic surfaces. Human cell lines may mimic the *in vivo* cell behavior and can be used for *ex vivo* biofilm assays. *Ex vivo* models also include biofilm growth on natural tissues in a minimally altered environment, offering more strictly controlled experimental conditions than those of *in vivo* models, which allows more detailed studies. The *ex vivo* models also facilitate studies of biofilm association with virulence, or therapeutic assays for experimental antibiofilm treatments and biofilm inhibition (Salas-Jara et al., 2016; Magana et al., 2018).

One environmental condition to which *Hp* is reactive is the endogenously produced quorum-sensing molecule autoinducer-2 (AI-2) that *Hp* senses as a chemorepellent. Anderson et al. (2015) studied the role of AI-2 chemotactic responses during *Hp* biofilm formation on biotic and abiotic surfaces. In the biotic assay, they adapted an *in vivo* model, with conditions similar to those previously described (Tan et al., 2011) using polarized Madin Darby canine kidney (MDCK) epithelial cells seeded onto a transwell filter. Experimental inoculation of *Hp* strains resulted in the formation of microcolonies on MDCK monolayers, in an AI-2 chemotaxis- dependent manner; similar results were observed in an abiotic model. Salas-Jara et al. (2016) evaluated biofilm formation of two probiotic strains, *L. fermentum* UCO-979C and *L. casei Shirota*, on the surfaces of two gastrointestinal cell lines, AGS and Caco cells. Sterile glass coverslips were placed in a 24-well polystyrene plate. The coverslips were treated with poly-L-lysine at 1 mg ml^-1^ to improve cell adhesion. Subsequently *Lactobacillus* strains were inoculated and the highest biofilm density was observed 12 h later. The biofilm formed by either *L. fermentum* UCO-979C or *L. casei Shirota* strains inhibited the adherence of *Hp* ATCC 43504 to both cells lines. SEM images of the formed biofilms are shown in their paper (Salas-Jara et al., 2016).

### *In vivo* Biofilm Formation in Animals

A recent study evaluated the potential of isolates of *Hp* to form biofilm in the stomach of C57BL/6J mice model. Mice were infected through gastric gavage with 10^8^ UFC of *Hp* strain. Infected mice were examined after 1 and 2 weeks. One week after the last challenge, the mice were sacrificed. For examination, the stomachs were removed and fixed in 4% paraformaldehyde (PFA) and *Hp* biofilm was demonstrated by immunofluorescence and SEM (Attaran et al., 2016).

### Detection of Biofilm-Associated *Hp* in Human Gastric Mucosa

The first evidence of biofilm formation by *Hp* during colonization in human gastric mucosa was photographic documentation by Carron et al. (2006). Using endoscopically obtained biopsy specimens and SEM analyses, they demonstrated the presence of dense mature biofilm-associated bacteria, attached to the cell surface of *Hp*-positive specimens. *Hp*-negative specimens, in contrast, had smooth mucosa with little evidence of a bacterial community (Carron et al., 2006). SEM also was used to quantify bacterial biofilm density on human gastric mucosa. Among patients with peptic ulcer disease, surface area covered by biofilms was 97.3% in *Hp*-positive patients, as compared to only 1.64% in *Hp*-negative patients (Coticchia et al., 2006).

To assess the biofilm-associated *Hp* in gastric biopsy specimens it is necessary to use methods other than routine ancillary stains, such as immunohistochemistry and fluorescence *in situ* hybridization (FISH). Preservation of three-dimensional structure is critical in order to analyze the spatial organization of the gut microbiota relative to mucin, host tissue and luminal contents (Hasegawa et al., 2017). Therefore, one of the crucial steps for gastrointestinal pathological laboratory is the fixation and embedment. The most common fixative is formaldehyde, an aqueous fixative, but several authors report that this fixative results in collapse or loss of the mucus layer and a widely used protocol is to process samples with non-aqueous Carnoy fixation to preserve the mucus layer for detection of bacteria adherent to the mucosal surface (Swidsinski et al., 2005; Johansson and Hansson, 2012). Johansson and Hansson (2012) observed a thick inner mucus layer firmly attached to the intestinal epithelia in non-aqueous Carnoy fixative, in contrast with a thin streak of collapsed mucus of less than 1 μm on the same tissue processed with formaldehyde fixative. Using confocal microscopy, Hasegawa et al. (2017) compared preservation of three-dimensional structure of four different embedding media: paraffin wax; polyester wax; optimal cutting temperature (OCT) compound; and glycol methacrylate resin. They conclude that hydrophobic embedding materials as paraffin require organic solvents to remove embedding resin and redistribution, collapse or loss of luminal contents could have occurred. In summary, although all of the examined embedments were capable of producing two-dimensional images, only glycol methacrylate resin enabled both retention and visualization of the three-dimensional luminal distribution of bacteria and food particles (Hasegawa et al., 2017).

The gastric surface mucous cells and gland mucous cells express secretory mucins, MUC5AC and MUC6, respectively. On the other hand, aberrant expression of the secretory mucin MUC2 in gastric mucosa is closely related to intestinal metaplasia and intestinal goblet cells (Ho et al., 1995; Reis et al., 1999; Babu et al., 2006; Matsuda et al., 2008). *Hp* may inhibit MUC5AC expression by the human gastric epithelium, and thus facilitate colonization. In contrast, increased MUC6 expression may help inhibiting colonization due to its antibiotic properties (Niv, 2015). FISH combines the molecular identification of bacteria with the direct visualization of the bacteria and the mucosa, which provides a significant advantage over culture, PCR, and histological methods alone (Swidsinski et al., 2005). Hasegawa et al. (2017) evaluated microbial FISH and two mucus labeling methods simultaneously to visualize microbial cells and host-derived mucus in intestinal sections following both Carnoy and PFA fixation and embedded in methacrylate or paraffin. Mucus was visualized with a fluorescent wheat germ agglutinin (WGA) and by indirect immunofluorescence using a primary antibody raised against mouse colonic mucin and both fixation methods allowed the visualization of intestinal mucus layers (Hasegawa et al., 2017).

**FIGURE 2:**
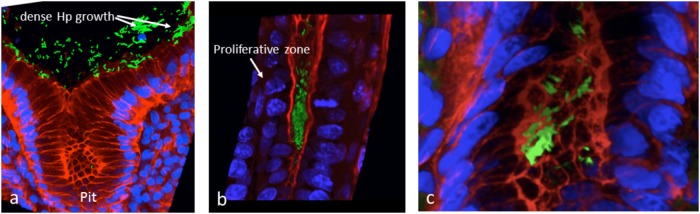
*In vivo* evidences suggestive of biofilm formation by *H. pylori* in the gastric glands of humans. **(a)** Large aggregates of *H. pylori* colonizing the surface of gastric glands; **(b)** *H. pylori* aggregates colonizing the neck of gastric glands, with proliferative cells; **(c)** colonies of *H. pylori* deep in the gland, in the vicinity of stem cells. *H. pylori* in green, actin in red and DNA nucleus in blue.

We attempted to obtain additional evidence of *Hp* biofilm formation in human gastric mucosa fixed in Carnoy, using FISH and *confocal laser scanning microscopy* (CLSM). We showed that *Hp* colonization is not limited to the surface of the human gastric glands, but can colonize deep in the glands, particularly in the region of the neck where cells are in constant proliferation and interestingly, also at the bottom of the glands, in intimate contact with the stem cells (Sigal et al., 2015). The identification of colonization of niches deep in the glands was possible because we studied large pieces of stomach coming from patients subjected to bypass surgery for control of weight. Using the same approach, we have confirmed the ability of *Hp* to form large aggregates suggestive of biofilm formation in the surface of the glands (Figure 2A), in the neck in close contact with proliferative cells and deep in the glands (Figure 2B), in the vicinity of stem cells (Figure 2C). In these studies, *Hp* was stained with antibodies specific to the bacteria, and confirmed the observed *in vivo* microbial aggregates were formed exclusively by *Hp*. However, it was not determined whether these aggregates were embedded in host mucins, bacterial EPSs or their combination. The challenge still remains to study the nature of the biofilm formation by *Hp in vivo*, in the gastric mucosa of humans and its role in the decades-lasting persistent colonization. The mechanisms of interaction of this biofilm-communities with the gastric mucosa at different niches in the glands may help us understand why in a few cases (<2%) the outcome of this interaction may be a severe disease such as gastric cancer. In particular, our observation of large aggregates of *Hp* growing in intimate contact with stem cells raises the possibility of damage to these cells as a result of direct interaction with the bacteria.

### Quantification of Mucosal Biofilms in Clinical Specimens

Quantification of mucosal biofilm bacteria is rather complex, and it has been performed almost exclusively for gut mucosal biofilms (Swidsinski et al., 2005; Dejea et al., 2014), Swidsinski et al. defined three distinct microscopic fields of the biofilm: (1) adherent bacteria (±1 μm of the epithelial border); (2) mucus-scattered bacteria present within mucus next to the epithelial surface; and (3) mucus ceiling bacteria present in the outer portions of the mucus, at least 10 μm away from the epithelial surface, and found marked increases in fields (1) and (3) for patients with inflammatory bowel disease, using several arithmetic assumptions (Swidsinski et al., 2005). Their data suggest that the average biofilm density in healthy gut mucosa is in a range of 1–5 × 10^8^ per ml (Swidsinski et al., 2005; Dejea et al., 2014), which reflects the fact that the inner mucus layer of normal human colon is impenetrable to bacteria (Matsuo et al., 1997; Johansson et al., 2008; Johansson et al., 2014). This number in healthy gastric mucosa may be even lower due to the high acidity, but to date, exact quantitation of *in vivo* biofilm in human stomach is lacking. We would need adjustment to the mathematical formula and bacterial probes used for gut biofilms in order to quantify *Hp* gastric biofilm.

## Genetic Regulatory Pathways of *Hp* Biofilm Formation

### Candidate Genes Involved in Biofilm Formation

Yonezawa et al. (2010) and Wong et al. (2016) report substantial *Hp* strain variability identifying strong biofilm producers and poor biofilm producers in both reference strains as well as clinical isolates. These strain differences are likely to reflect variability in bacterial genes involved in biofilm formation and may be important for targeted intervention to reduce consequences from such infection. Existing literature suggests that three essential traits are required for bacteria to form and grow in biofilm *in vivo*: (1) inter-bacterial communication, (2) coordinated movements, and (3) aggregation. Furthermore, genes that control bacterial shape to fit biofilm environment may have an additional role in biofilm formation and maintenance.

Inter-bacterial communication is mediated through quorum sensing (QS) system, which is mediated by production and release of chemical signaling molecules in response to cell density and other physiological conditions (Elias and Banin, 2012; Gölz et al., 2012; Majumdar and Pal, 2017). These signaling molecules alter the expression of bacterial QS dependent genes to induce bacterial phenotype changes in virulence, motility, chemotaxis and biofilm formation, thus contributing to adaptation and colonization to the host (Elias and Banin, 2012; Gölz et al., 2012; Majumdar and Pal, 2017). To date, multiple systems based on different groups of molecules have been identified, some very bacteria-specific and others more general. Among those, signaling molecules called autoinducers (AI), AI-2 system, are most universally used by many bacteria, including *Hp* (Elias and Banin, 2012; Gölz et al., 2012; Majumdar and Pal, 2017). Another molecule produced by multiple bacterial families is N-acylhomoserine lactone (AHL) (He and Zhang, 2008; Elias and Banin, 2012; Papenfort and Bassler, 2016), although there have been no data to support that *Hp* utilizes the AHL-based QS. The other group of molecules more recently discovered is termed diffusible signal factor (DSF) that consists of fatty acids of various chain lengths and branching and is known to be used by several groups of bacteria (He and Zhang, 2008; Elias and Banin, 2012; Papenfort and Bassler, 2016). One well-characterized DSF is 2-(Z)-tetradecenoic acid (TDA) produced by the xylem-limited plant pathogen *Xylella fastidiosa* (Ryan and Dow, 2011; Ryan et al., 2015). Recently, Yamashita et al. reported two self-growth-inhibiting compounds in *Hp* and one of them was identified as 7-(Z)-TDA (Yamashita et al., 2015). Although it has not been reported as a DSF, 2-(Z)-TDA is a positional isomer of 7-(Z)-TDA. Thus, 7-(Z)-TDA probably also acts as a signaling molecule to control the cell density of *Hp*. Currently, it is not known which *Hp* genes are responsible for synthesizing this compound. There are no other *Hp*-specific QS molecules characterized thus far. Several studies have also revealed that signaling pathways induced by QS molecules are followed by the phospho-relay cascade transmitted from the membrane-bound receptors to the cytosolic second-messenger system (Bharati and Chatterji, 2013; Wolska et al., 2016; Jakobsen et al., 2017). Second messengers based on mono (cAMP and cGMP) and di-cyclic or modified nucleotide (ppGpp, c-di-GMP and c-di-AMP) play a crucial role in transmitting the signals received from the surface receptor to the target molecule in the cell. Cyclic dimeric guanosine monophosphate (c-di-GMP) has evolved as a key activator of biofilm formation in almost all bacteria (Römling and Balsalobre, 2012; Bharati and Chatterji, 2013; Wolska et al., 2016; Jakobsen et al., 2017). c-di-GMP is synthesized by the GGDEF domain proteins and degraded by the unrelated EAL and HD-GYP domain proteins. The c-di-GMP signaling network is the most complex secondary signaling system found in bacteria with more than 100 c-di-GMP-metabolizing proteins in some species. This signaling network is especially prominent in γ-Proteobacteria, including the major human pathogens *Pseudomonas aeruginosa, Salmonella typhimurium, Escherichia coli*, and *Vibrio cholerae*, but little is known about this network in *Hp* (Römling and Balsalobre, 2012).

After bacteria reach a suitable niche to live, guided by chemotaxis, bacterial congregation requires adhesive materials, known as EPS. EPS are mostly produced by bacteria and consist of polysaccharides, proteins, enzymes, nucleic acids, lipids, other biopolymers, extracellular bacterial structures such as flagella, pili, fimbriae, and OMV (Flemming and Wingender, 2010). Interestingly, in contrast to many other bacteria, proteins appear to be the central player in *Hp* biofilm matrix (Windham et al., 2018). Recent studies also indicate that these three processes are not only controlled by *Hp* protein-coding genes, but also by a number of non-coding small regulatory RNAs (Mika and Hengge, 2013; Fazli et al., 2014; Bak et al., 2015; Wolska et al., 2016). Finally, it is also noteworthy that host molecules may contribute to EPS and host-bacterial co-aggregation. Specifically, secretory immunoglobulin A (IgA) has been shown to facilitate biofilm formation by normal gut flora in human tissue culture and by *Escherichia coli* in an intestinal cell line (Randal et al., 2003), as well as in a mouse model mono-infected with commensal *Bacteroides fragilis* (Donaldson et al., 2018). Interestingly, a higher systemic IgA response has been reported in mice infected with a high biofilm producing *Hp* than in those infected with low biofilm producing *Hp* strain, although mucosal secretory IgA levels were not quantified (Attaran et al., 2016).

### Biofilm-Associated *Hp* Genes

#### Inter-bacterial Communication (QS Molecules)

AI-2 is produced as a metabolic byproduct of the reaction carried out by LuxS, which cleaves S-ribosylhomocysteine, producing homocysteine and 4,5-dihydroxy-2,3-pentanedione (DPD) and AI-2 as a metabolic byproduct of this reaction. DPD undergoes rapid dehydration and cyclization, existing in equilibrium as several molecules collectively termed as AI-2 (Schauder et al., 2001). LuxS uses methionine as a reduced sulfur source in the processes of *de novo* cysteine biosynthesis pathway. The *luxS* gene maps within an operon encoding *cysK, metB*, and *luxS*, which are necessary for the *de novo* cysteine biosynthesis pathway (Doherty et al., 2010). Some *luxS* mutants show reduced motility, and present a reduced number and length of each flagella. These differences result from a reduced transcription of genes of the flagella biosynthesis pathway (such as *flaA, flgE, motA, motB, flhA*, and *fliI* but not *flaB* genes) (Osaki et al., 2006; Rader et al., 2007; Shen et al., 2010). Gölz and colleagues demonstrated that a normal motility could be restored by adding AI-2. All described defects affecting motility of *Hp luxS* mutants were restored by the addition of AI-2 or DPD (Gölz et al., 2012). In *Hp*, AI-2 functions as a signaling molecule up-stream of the flagellar regulator *flhA*. High amounts of AI-2 increases motility, but it also reduces biofilm formation by *Hp*, thereby leading to colonization of niches with a small number of bacteria, but with a better provision of nutrients. AI-2 influences flagellar gene expression up-stream of *flhA* (Tsang and Hoover, 2014) and functions as chemorepellant via TlpB (Gölz et al., 2012). Both mechanisms obviously contribute to regulation of biofilm formation vs. planktonic growth, which in turn promotes bacterial colonization and persistence in the stomach (Rader et al., 2011).

The *Hp* genome carries few regulatory elements. Three of those are Fur, regulating the ferric uptake; NikR, regulating the response to the presence of nickel response regulator; and ArsRS, a two component system, which responses to an acid environment (Danielli and Scarlato, 2010). In particular, ArsR is essential for *in vitro* survival (Loh et al., 2010). Mutation studies of ArsS have been performed by mutating *arsR* into a non-phosphorylatable form (ArsR-D52N mutant). Involvement of ArsS in biofilm formation has been suggested by proteomic analysis and because ArsS mutants have a significant increase cellular aggregation and adherence to the flask at the air-liquid interface. Further studies showed that strains carrying ArsRD52N mutation or a combination of *nikR* and *arsS* deletions had a quicker transition to the coccoid form. Similar to biofilm formation, the transition to the coccoid form is known to happen in case of environmental stressors, such as nutrient deprivation. In summary, the data of these studies suggest that the lack of ArsRS system increases stress response, leading to an increased and faster biofilm formation. The simultaneous deletion of *arsS* and *nikR* genes decreases the normal *Hp* functionality and results in increasing of the coccoid forms and increased biofilm formation (Servetas et al., 2016). This hyper-biofilm forming phenotype of *arsS* mutants is found to be mediated by an outer membrane protein, *homB* (Servetas et al., 2018).

#### Chemotaxis That Guides Movement to Reach the Right Position

In the colonization of gastric mucosa by *Hp*, coordinated movements are required to reach and position in the right topology of the stomach horizontally and vertically to form stable biofilms, guided by chemotaxis and powered by flagella and pili (Harshey, 2003; Howitt et al., 2011; Persat et al., 2015). In fact, aflagellated *Hp* mutants exhibit impaired biofilm formation (Hathroubi et al., 2018b). *Hp* species regulate their motility by chemotactic signaling systems, which allow the bacteria to follow favorable chemical gradients in their host environment. A number of transcriptional factors are known to be involved in these processes (De la Cruz et al., 2017). For chemotactic signal transduction, four different groups of proteins are necessary: (1) chemoreceptors, (2) core signal proteins, (3) accessory proteins, and (4) flagellar switch proteins (Lertsethtakarn et al., 2011). In *Hp*, the chemotactic behavior to low pH is dependent on the chemoreceptor TlpB (Croxen et al., 2006). A *luxS* mutant of *Hp* strain G27 showed a reduced stopping frequency in liquid media, which was restored by the addition of AI-2 or DPD (Rader et al., 2011). Analyzing the chemotactic behavior of double and single mutants, these authors confirmed that AI-2 is perceived as a chemorepellant signal via TlpB. In fact, an *Hp* strain deficient for the chemoreceptor TlpB failed to move away from a source of synthetic DPD and did not display increased stopping behavior upon addition of synthetic DPD. These behaviors were restored upon genetic complementation of the *tlpB* gene. *Hp* chemoreceptor TlpB is required for recognition of AI-2, which confirms its role in negative pH taxis (Rader et al., 2011). ChePep mediates another signaling system that controls the flagellar switching in a chemotaxis signaling protein-dependent manner. ChePep localizes to the flagellar pole of *Hp*, and chePep mutants show aberrant flagellar rotation, although flagella are correctly assembled and motile. In fact, ChePep does not control directly flagellar switching, but functions as a chemotaxis regulator, as it has been shown by studying *Hp* chemotactic behavior in a pH gradient. *Hp* cells increase their turning frequency in presence of a chemorepellent (i.e., acid) because of a predicted increase in phosphate signaling in the chemotaxis system. ChePep mutants constantly switch flagellar rotation also in normal culture condition and respond to acid condition by increasing their turning frequency. This behavior can be explained by high levels of phosphorylated CheY protein as obtained in some enteric bacteria by the mutation of CheZ and CheB that normally function as chemotaxis regulators by reducing the phosphorylation state of CheY (Howitt et al., 2011).

#### Extracellular Matrix, Outer Membrane Vehicle, and Adhesins Involved in Biofilm Formation

The Tol Pal gene cluster has been extensively studied in other organism such as *Escherichia (E) coli* and its role in the formation of OMV and in bacterial cell integrity is well known. *Hp* genomes encode homologous genes to *E. coli tolB* and *pal* (*HP1126* and *HP1125*, respectively), but no *Hp* homolog for *tolA* has been described (Turner et al., 2015). Moreover, *Hp* possesses two coding regions (*HP1127* and *HP1128*), with no known homologs in the databases, and other two coding sequences, *HP1130* and *HP1129*, putative homologs of *tolQ* and *tolR*, respectively. *Hp tolB* mutants displayed extensive “blebbing” and had very few flagella, which were markedly shorter in length and appeared to form clumps, suggesting a division defect. The morphology of the double mutant *tolB*-*pal* was not very different to that of wild type (WT) bacteria, although mutants lacked flagella completely. This phenotype could be due to the fact that the peptidoglycan-associated outer membrane proteins, including those of the Pal family, share sequence homology in the C-terminal region with the *E. coli* motility protein, MotB (Mot and Vanderleyden, 1994). This region of MotB is particularly important in anchoring the flagella structure to the peptidoglycan. It is therefore possible that any disruption of either the TolB or Pal protein may affect the functioning of the Tol-Pal complex as a whole, thereby altering flagella synthesis or hindering the anchoring of the flagella to the peptidoglycan layer. Furthermore, *Hp* ΔtolB and Δpal mutants produce >600- and 22-fold more OMVs than WT bacteria, suggesting a strong co-regulation of flagella and OMV (Turner et al., 2015).

Efflux pump functions in the formation of biofilms and multidrug resistance (Soto, 2013). *gluP* has been shown to be involved in the biofilm formation and multidrug resistance of *Hp* and the expression of *gluP* is upregulated by SpoT, which is a known global regulator (Ge et al., 2018). In addition to *SpoT*, there are other genes involved in both biofilm formation and multidrug resistance in *Hp*, which is mostly mediated through an increased expression of RND efflux pump genes (Yonezawa et al., 2013; Attaran et al., 2017). Furthermore, The *gluP* encodes a glucose/galactose transporter that belongs to the major facilitator superfamily of the efflux pump machinery (Tomb et al., 1997), which is mainly responsible for the physiological uptake of sugars, such as D-glucose (Psakis et al., 2009). Its inactivation has also the effect to alter the biofilm formation because glucose is a component of polysaccharides (Limoli et al., 2015) that are part of biofilm matrix (Ge et al., 2018).

Using nuclear magnetic resonance, Yang et al. (2011) identified mannose-related proteoglycans (proteomannans) as a major contributor to *Hp* EPS in *in vitro* culture. They detected the presence of NapA protein upregulation in the biofilm, suggesting a mechanism to increase adhesiveness of *Hp* biofilm. An isogenic mutant of *napA* revealed an altered biofilm structure with reduced aggregates, when compared to the WT. Adhesins are another class of genes involved in biofilm and the *Hp* genome contains two adjacent homolog genes, *alpA-alpB*, annotated as *omp20-omp21* and *hopC-hopB* in the 26695 and G27 genomes, respectively, that may be involved in biofilm formation. A recent study (Yonezawa et al., 2017) compared OMV protein profiles between a *Hp* strain and its spontaneous weak biofilm-forming mutants and demonstrated that AlpB was an important protein for biofilm formation. *alpB* mutants derived from various strains have also been shown to be incapable of inducing cell aggregation (Senkovich et al., 2011; Yonezawa et al., 2017). Furthermore, these studies suggest that particularly the variable region of *alpB* is involved in the attachment to various substrates, including gastric cells.

**FIGURE 3:**
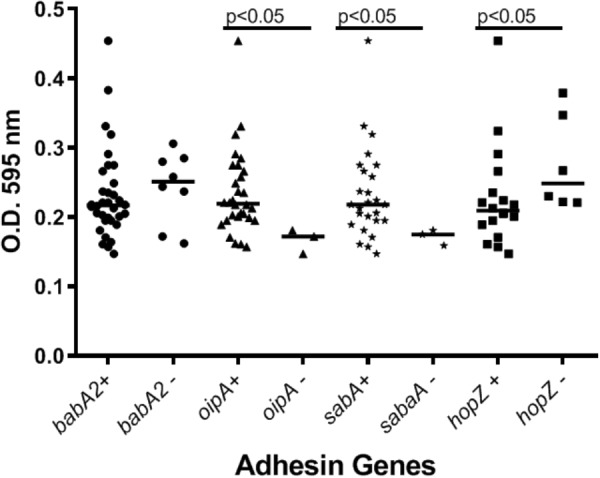
The presence of adhesins in *H. pylori* strains may influence the formation of biofilm. See text for details on the assay.

**FIGURE 4:**
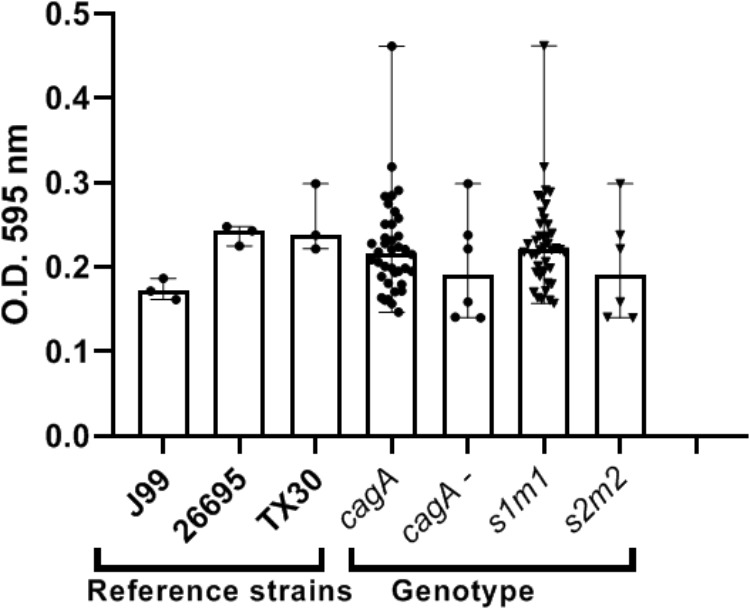
*cagA* or *vacA* genotypes did not influence the capacity to form biofilm by *H. pylori* strains. See text for details on the assay.

Our preliminary studies on several known *Hp* virulence genotypes and biofilm formation *in vitro* support potential involvement of some adhesins. We tested a total of 45 clinical isolates from Mexican patients, including 15 each for non-atrophic gastritis, intestinal metaplasia and gastric cancer, using 96-well polystyrene microtiter plates. Each well was filled with 135 μl of Brucella broth. The formation of biofilm was started by inoculating 15 μl of pre-cultured bacterial suspension with 3 × 10^8^ CFU/ml into each well. The cultures were incubated under microaerobic conditions during 48 h, at 37°C and 10% of CO_2_. Uninoculated Brucella broth served as a negative control. After incubation, the supernatant was removed, the plates were washed 3 times with PBS and dried for 30 min. One hundred and fifty microliter of 0.1% crystal violet was added to each well and the plate was incubated at room temperature for 20 min. The excess crystal violet stain was removed, and the plate washed 3 times with PBS. The crystal violet staining biofilm was extracted with 150 μl of ethanol (70%) and measured at OD 595 nm. The assay was done 3 times and the final results were the mean of absorbance values. We tested strains positive or negative for adhesins reported important for *Hp* colonization. Strains lacking either *oipA* or *sabA* showed a significantly reduced ability to form biofilm. In contrast, strains lacking *hopZ* were in fact more efficient producing biofilm (Figure 3), and we found no differences between strains with or without the *babA2* gene. These results need to be confirmed with the study of additional strains, including isogenic strains mutated in the gene under study. On the other hand, our initial results indicate that *cagA* and *vacA* genotype may have no influence in the intensity of biofilm formation (Figure 4); formation of biofilm was similar in both, *cagA*-postive strains (J99 and 26695) and *cagA*-negative strains (TX30). Formation was also similar between *vacA* s1 and s2, or m1 and m2 strains; although we observed a tendency for reduced biofilm formation by m2 *Hp* strains (Figure 4). These observations ensure that the observed association with some adhesines was not mediated through the correlation with the presence of *cagA* and *vacAs1m1* genotypes.

#### Genes Regulating the Shape of *Hp*

The morphological transition of *Hp* leads from a spiral rod–shaped organism to a coccoid organism by changes in the peptidoglycan of the bacterial cell wall, These modifications are mediated by AmiA (Chaput et al., 2006, 2016). Moreover these changes influence the ability of the coccoid form to escape the immune system. Studies have shown that peptidases Csd1-4 and Csd6, as well as potential regulators Csd5 and CcmA are required to tailor the PG layer to generate the helical cell shape characteristic of *Hp* (Sycuro et al., 2010, 2012, 2013). Csd4 cleaves the gamma-D-Glu^2^-*m*DAP^3^ bond of the muramyl tripeptide to produce the muramyl dipeptide (member of M14 metallopeptidase family, Zn^2+^-dependent). Csd3 (also known as HdpA, member of the M23 metallopeptidase family) has D,Dendopeptidase and D,D-carboxypeptidase (D,D-CPase) activities. Csd6 (*HP0518)* belongs to the peptidoglycan trimming pathway and also influences the cell shape of *Hp*. Disruption of the *csd6* gene by transposon-induced mutation or its deletion resulted in a straight rod shape and in an increase in tetrapeptide-containing muropeptides (Kim et al., 2015).

More recently Blair et al. (2018) studied the mechanisms by which Csd5 promotes helical cell shape. It has been shown that the N-terminal cytoplasmic (NT) and transmembrane (TM) domains, together with a C-terminal SH3 domain in Csd5 are each required to promote helical shape. Csd5 interacts directly with peptidoglycan via its C-terminal SH3 domain, whereas the N-terminal transmembrane domain promotes interactions with CcmA, MurF that catalyzes the synthesis of a PG precursor, and F_1_F_0_ ATP synthase. The recognition of the interaction of Csd5 protein with CcmA, a known cell-shape protein and putative cytoskeletal bactofilin, and with MurF, a known cell elongation factor, was unexpected but not surprising given the connections between PG synthesis, cell shape and intermediate filament proteins in helical organisms. Another study investigated the cholesteryl glucosides (CGs), one of the major components of the cell wall, by the deletion of the *hp0421* gene, which encodes cholesteryl α-glucoside transferase that integrates (CGs) into the cell wall of *Hp.* This determines a deficiency of cholesterol, an alteration in the morphology and shape (“c”-shaped cells were prevalent), and of cell walls components like LPS, which leads to less virulent strain and an increasing of susceptibility to antibiotics (Qaria et al., 2018).

#### *Hp* Genetic Variabilities in Biofilm-Associated Genes

**FIGURE 5:**
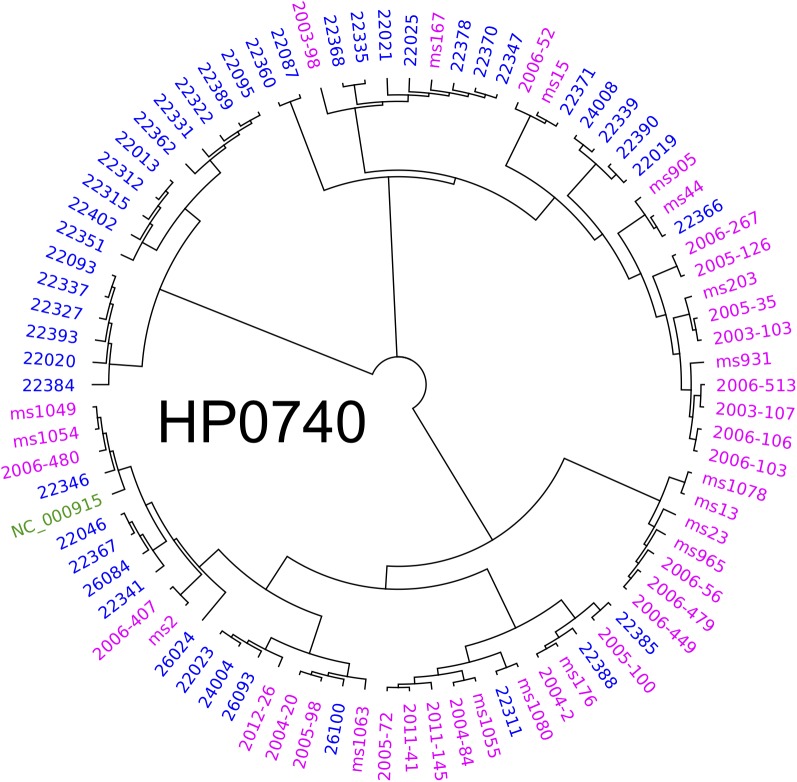
Phylogenetic analyses of *HP0740* for 74 Mexican and Colombian strains. The alignment and graphics was produced with Geneious and graphically modified with Inkscape software.

To elucidate the diversity of biofilm-associated genes among *Hp* strain, we used whole genome data (Muñoz-Ramírez et al., 2017) of 74 Latin American *Hp* strains to analyze 33 genes reported to be involved in biofilm formation (Table 1). This analysis revealed a mean variation of 6.78% in the amino-acidic sequence, ranging from a minimum of 0.39% in *motA* gene (*HP0815*, 1 non-synonymous variant in 258 amino acids) to a maximum of 23.12% in *chePep* gene (*HP0322*; 318 non-synonymous variants in 506 aa). For *luxS* (*HP0105*) that encodes an enzyme to produce AI-2 (He and Zhang, 2008; Gölz et al., 2012; Wolska et al., 2016), we found 10 non-synonymous variants with a variant frequency >10% on a total of 453 bp. Further phylogenetic analysis of the 33 genes in the 74 *Hp* strains did not show evidence of geographical clustering by country for most of these genes with the only exception of the *murF* gene (*HP0740*), which clearly formed separated clusters for Mexico and Colombia (Figure 5). This result would suggests that *murF* is under constant positive selection to adapt to the human host population they are colonizing. Whether this diversity in *murF* contributes to differential gastric cancer risk in these two populations is something that deserves further studies.

**Table 1:** Variation analysis in *Hp* biofilm related genes.

Gene name	26695 gene code	Non-synonymous variants > 1% frequency	Non-synonymous variants > 10% frequency	Gene length (bp)	Potential pathways and functional categories (see footnotes)/additional references for genes not referred in the text
*luxS*	*HP0105*	36	10	453	(1)
*arsR*	*HP0166*	18	18	683	(1)
*aibB*	*HP0473*	114	36	753	(1), (2)/(Anderson et al., 2015)
*aibA*	*HP0298*	120	34	1658	(1), (2) /(Anderson et al., 2015)
*fur*	*HP1027*	18	4	454	(1), (2)
*rpoN*	*HP0714*	135	29	1247	(2)
*tlpB*	*HP0103*	224	33	1700	(2)
*fliA*	*HP1032*	61	20	804	(2)/(De la Cruz et al., 2017)
*flgR*	*HP0703*	45	17	1147	(2)/(De la Cruz et al., 2017)
*chePep*	*HP0322*	322	318	1518	(2)
*cheW*	*HP0391*	23	5	498	(2)
*cheY*	*HP1021*	69	7	897	(2)
	*HP1067*	21	2	375	
*cheA*	*HP0392*	61	22	2412	(2)
*flaA*	*HP0601*	52	8	1571	(2)
*flgE*	*HP0908*	30	8	1818	(2)
	*HP0870*	27	6	2160	
*motA*	*HP0815*	32	1	774	(2)
*motB*	*HP0816*	41	11	779	(2)
*flhA*	*HP1041*	42	12	2202	(2)
*nikR*	*HP1338*	52	8	447	(3)
*tolB*	*HP1126*	104	20	1255	(3)
*HP0840/JHP0778*	*HP0840*	42	13	1024	(3)/(Burrows et al., 2000)
*futA*	*HP0379*	193	196	1278	(3)/(Wong et al., 2016)
*futB*	*HP0651*	218	241	1431	(3) /(Wong et al., 2016)
*homD*	*HP1453*	278	84	2303	(3) /(Wong et al., 2016)
*napA*	*HP0243*	29	5	435	(3)
*amiA*	*HP0772*	101	27	1323	(4)
*ccmA*	*HP1542*	24	5	411	(4)
*csd1*	*HP1543*	103	13	948	(4)
*csd2*	*HP1544*	87	27	928	(4)
*csd4*	*HP1075*	127	39	1317	(4)
*csd5*	*HP1250*	104	27	579	(4)
*csd3*	*HP0506*	200	33	1281	(4)
*csd6*	*HP0518*	100	20	983	(4)
*murF*	*HP0740*	162	54	1485	(4)
*cgt*	*HP0421*	53	53	1170	(4)


*Functional categories: (1) Sensing and communications, quorum sensing molecules, (2) Movement/positioning, chemotaxis, flagella, (3) Extracellular matrix production, polysaccharide metabolism, outer membrane vehicle synthesis, (4) Cell shape control.*

## Functional and Morphological Changes Associated With Biofilm Formation

### Growth Rate

Slow growth rate has been recognized as one of the major contributing factors to antibiotic resistance of biofilm-associated bacteria (Stewart, 2015). Matured biofilms are known to contain variable fractions of slow growing or non-dividing metabolically inactive cells, which gives rise to persistent colonization (Lewis, 2005; Kostakioti et al., 2013). *In vitro* mono-microbial biofilm studies demonstrated that *Streptococcus mutans* biofilms grew about 35% slower than planktonic culture before the stationary phase (Welch et al., 2012). Using transcriptional profiles, Folsom et al. estimated that *Pseudomonas aeruginosa* grew at just 10% of planktonic growth rate in the stationary biofilms (Folsom et al., 2010). Applying an agarose gel as artificial EPS, Pabst et al. confirmed that the growth rate of *Staphylococcus aureus* in the stationary biofilms was also about 10% of that of planktonic culture (Pabst et al., 2016). Altered gene expression leading to reduced transcription and to translation and DNA replication arrest, has been identified as molecular characteristics of the stationary biofilm-associated cells (Lewis, 2005; Stewart, 2015). Others suggest that bacterial synthesis of antibacterial compounds against own or neighboring organisms (Elias and Banin, 2012; Yamashita et al., 2015) may also contribute to the slow growth of biofilm-associated bacteria. To date, however, there have been no direct *in vitro* or *in vivo* data to support that *Hp* forming stationary biofilms exhibits a reduced growth rate.

### Metabolism

Biofilms are formed by groups of cells in different states, growing or non-growing and metabolically active or inactive in variable fractions, depending on maturity and on chemical gradients (O_2_ and nutrients) of the biofilms (Stewart, 2015; Flemming et al., 2016). It has been postulated that dormant non-growing metabolically inactive cells reach these metabolic states in order to reduce cell permeability and protect themselves against oxidative stress (Stewart, 2015). Bacterial cells in stationary biofilms have also been reported to increase synthesis of pili, fimbriae and exopolysaccharides as well as lipopolysaccharide modification and production of stress-response enzymes (Prigent-Combaret et al., 1999; Folsom et al., 2010). Wong et al. recently profiled metabolites of high and low biofilm forming *Hp* strains from Malaysia using liquid chromatography/quadrupole time-of-flight mass spectrometry (Wong et al., 2018). The study compared four high and four low biofilm forming strains cultured up to 3 days in duplicate. Interestingly, low-biofilm-formers produced more metabolites than high-biofilm-formers, consistent with lower overall metabolic activities in biofilm-associated bacteria. Further analysis indicated that the metabolites significantly lower in high biofilm producers belonged to major categories of lipids, with an important role in bacterial-bacterial communications, and metabolites involved in prostaglandin synthesis (Wong et al., 2018).

### Morphology

A morphological change described as small colony variants (SCVs) has been linked to various biofilm associated infections, such as cystic fibrosis, osteomyelitis and device-related infections (Proctor et al., 2006). Since its first description for *Salmonella enterica serovar typhi* (*S. typhi*) almost 100 years ago, SCVs have now been reported for a wide range of bacterial genera and species, including *Staphylococci, Escherichia coli, Pseudomonas aeruginosa, Vibrio cholerae, Shigella* spp., *Lactobacillus acidophilus*, and *Neisseria gonorrhoeae* (Proctor et al., 2006). Currently, the connection between the SCV phenotype and persistent, recurrent infections has become a hot topic in clinical microbiology. Phenotypically, the size of SCVs are about one tenth of that in regular colonies, with a slow growth rate, atypical colony morphology and unusual biochemical characteristics and are less susceptible to antibiotics than their wild-type counterparts. An altered cellular morphology in the cell wall structure and the emergence of intercellular EPS have also been demonstrated for SCVs from *Staphylococcus aureus*. These characteristics have been associated with mutations in the *nupC* gene that encodes a protein involved in thymidine uptake and in the *thyA* gene that encodes thymidylate synthase (Proctor et al., 2006). A group of Norwegian researchers reported spontaneous appearance of *Hp* SCVs, although they did not clarify its size relative to normal colonies (Bukholm et al., 1997). This variant was induced by acid exposure and showed enhanced adherence and invasion to epithelial cells (Bukholm et al., 1997; Tannæs et al., 2001). It was found that these morphological changes are a consequence of a shift in cell wall lipid composition, specifically, an increase in lysophospholipids, due to phase variation in the *pldA* gene, resulting in production of an active form of the outer membrane phospholipase A (OMPLA) (Bukholm et al., 1997; Tannæs et al., 2001). Another colony variant associated with biofilm is the rough colony variant arising from smooth colonies of *P. putida* (Hansen et al., 2007; Elias and Banin, 2012). This was caused by two independent mutations in *wapH (PP4943*), a gene involved in lipopolysaccharide (LPS) biosynthesis (Hansen et al., 2007). Rough/smooth phenotype variants due to altered LPS composition were also observed for *Hp*, yet its association with biofilm formation and virulence is unclear (Bertram-Drogatz et al., 1999).

At the cellular level, the best-described morphological change in *Hp* associated with biofilm *in vivo* is coccoid transformation (Carron et al., 2006; Coticchia et al., 2006; Cellini et al., 2008). Coccoid transformation is considered a common feature of Gram-negative rod bacteria under conditions of stress (Andersen and Rasmussen, 2009; van Teeseling et al., 2017; Krzyzek and Gosciniak, 2018). *In vitro*, coccoid forms of *Hp* can be induced by prolonged culture or use of suboptimal levels of antibiotics (Krzyzek and Gosciniak, 2018; Poursina et al., 2018). The *Hp* coccoid forms have been defined as viable but non-culturable (VBNC), thus undetectable by conventional cultures, and are known to have an increased capacity to aggregate into monomicrobial clusters embedded in thick EPS (Cellini et al., 2008; Cellini, 2014; Krzyzek and Gosciniak, 2018). In many ways, VBNC coccoid *Hp* resembles the characteristics of persister cells documented in biofilms of other bacteria (Lewis, 2005; Pinto et al., 2015) and they can survive up to 1 year just in fresh water (Cellini, 2014; Percival and Suleman, 2014). An accumulation of N-acetylglucosaminyl-N-acetylmuramyl–l-Ala–d-Glu in PG of the cell wall (Costa et al., 1999), catalyzed by AmiA (PG hydrolase), has been described to precede this morphological transformation and also to lead to immune evasion by escaping from NOD1 detection (Chaput et al., 2006). Yet, since PG dictates bacterial cell shape (Rubin and Trent, 2013; van Teeseling et al., 2017) and since many other PG remodeling enzymes have been described (Caccamo and Brun, 2018), genes other than *amiA* are likely to be involved in this process. In fact, genes encoding the enzymes, *pgp1* and *pgp2 for Campylobacter* spp, and *csd1, csd2, csd3, csd4, csd6, murF* and related non-enzymatic proteins, *csd5* and *ccmA* for *Hp* have been identified to be critical in the maintenance of helical shape (Sycuro et al., 2010, 2012, 2013; Kim et al., 2015; Esson et al., 2016; Blair et al., 2018) as detailed in the previous section. In addition, coccoid *Hp* harvested *in vitro* from clinical isolates overexpressed *spoT*, a global transcriptional regulator for stress responses (Poursina et al., 2018). A homologous gene in *Mycobacterium smegmatis* has been linked to its cell shape control (Gupta et al., 2016) and a *Hp spoT*-mutant exhibits a reduced ability to form biofilm (Ge et al., 2018). To date, despite these growing observations, it remains to be elucidated whether coccoid transformation indeed prompts biofilm formation, or whether biofilm formation promotes *Hp* coccoid transformation.

### Virulence Factors

It is yet unknown whether biofilm-associated bacteria exert virulence more potently than those living planktonically. There are several factors that affect virulence of biofilm-associated bacteria. As discussed above, the stage (maturity) of biofilms, chemical composition (O_2_, acidity and nutrients gradients) and microbial (mono- vs. poly-biofilms) environment within biofilms are important determinants of bacterial gene expression (Lewis, 2005; Folsom et al., 2010; Stewart, 2015). In addition, how to quantify or compare virulence is not so straightforward, because life expectancy of bacteria living in biofilms and that of planktonic bacteria are different, as the former is expected to live longer causing persistent infection (Lewis, 2005; Pinto et al., 2015). Furthermore, even if biofilm-associated bacteria express lower virulence per time compared with planktonic bacteria, cumulative effects on host cells may be higher. In addition, aggregative nature of biofilm-forming bacteria may enhance virulence through increased adherence to host cells, even if the same amount and potency of virulence molecules are produced or released. Part of biofilm-associated virulence is also derived from surrounding EPS, not bacterial cells themselves. EPS contains secreted enzymes, toxins and outer membrane vehicles (Flemming and Wingender, 2010; Hathroubi et al., 2018a) and can activate neutrophils without opsonization to induce inflammatory reactions and exert cytotoxicity (Meyle et al., 2012; Fulsundar et al., 2015; Susanne et al., 2015; Kim et al., 2016; Nascimento et al., 2016).

Pinto et al. reviewed virulence of VBNC bacteria predominantly found in biofilms (Pinto et al., 2015). They concluded that most bacteria continue to express their virulence and toxin genes, but expression levels are downregulated or protein secretion may not be detectable. However, altered gene/protein expression seems to vary with types of bacteria, types of virulence genes, or co-colonization with other bacteria. For example, when *Pseudomonas aeruginosa* and methicillin-resistant *Staphylococcus aureus* (MRSA) were grown together as a mixed culture, biofilm development was accelerated and production of *P. aeruginosa* exotoxin A was increased by 1839-fold in comparison to their respective monocultures (Goldsworthy, 2008). Moreover, *P. aeruginosa* exoproduct 4-hydroxy-2-heptylquinoline-*N-*oxide (HQNO) was found to stimulate *S. aureus* biofilm and SCV formation, leading to an increase in the expression of the fibronectin-binding protein A and a decrease in the expression of the α-hemolysin gene (Mitchell et al., 2010). It was also demonstrated that a spontaneous SCV of *Hp* released VacA and urease from the cells, while its parent large colony cells retain these toxins within the cells (Bukholm et al., 1997).

Other studies specific to *Hp* have attempted to identify differential virulence profiles between coccoid and helical/spiral forms. A proteomic study by Bumann et al. (2004) reported differential virulence profiles, i.e., *cagA*/*vacA* dominance in helical and *ureA/B* and *groEL* dominance in coccoid forms. The lack of CagA protein expression in coccoid *Hp* was consistent with an earlier report by Roe et al. (1999). However, a more recent high resolution proteomic study reached a different conclusion (Loke et al., 2016). When compared with helical forms, coccoid forms of *Hp* were found to express higher levels of proteins that are involved in virulence and carcinogenesis, such as secretion system machinery proteins, CagE, CagV, and YidC and proinflammatory proteins, such as OipA and Hps. Poursina et al. tested the mRNA expression of two *Hp* virulence genes, *babA* and *cagE* in coccoid forms harvested *in vitro* and concluded that both genes were expressed but in lower rates than those of helical forms. Also, coccoid forms of clinical isolates from peptic ulcer patients showed higher *cagE* expression than the reference *Hp* strain 26695, indicating the presence of strain variability (Poursina et al., 2013). More recently, Hirukawa et al. (2018) found that all forms of *Hp* (helical, coccoid and fragmented) expressed certain pathogenic proteins, including CagA, other components of the cag-Type IV secretion system (VirB7 and VirB9), the blood group antigen-binding adhesin BabA, and UreA, to similar levels. However, phosphorylation of CagA in gastric cancer cells was only seen by the helical form (Hirukawa et al., 2018). In contrast, Segal et al. (1996) reported, using the same cells but a different strain, that coccoid *Hp* was capable of binding and inducing cellular changes of the same sort as spiral *Hp*, including tyrosine phosphorylation of host proteins and that coccoid *Hp* induced a stronger cytoskeletal rearrangement than spiral *Hp*. Co-culture of a gastric non-cancer epithelial cell line with coccoid or helical forms of *Hp* revealed that helical *Hp* led to more severe inflammatory and apoptotic responses, while coccoid *Hp* maintained host cell in a high proliferation rate (Liu et al., 2006). Interestingly, in animal models, colonization efficiency of *Hp* mutants in coccoid associated genes, *aimA* and *spoT*, was markedly reduced (Sun et al., 2012; Chaput et al., 2016). Despite substantial contrasting findings in the observations, overall, accumulated data suggest that coccoid *Hp* retains virulence, can survive better *in vivo* and may contribute to carcinogenesis, while helical *Hp* can be more easily eliminated by acute host immunoinflammatory responses.

The toxin–antitoxin (TA) systems have emerged as important virulence factors in many pathogenic bacteria, which have been described to be beneficial in bacterial fitness, persistence, and virulence (Kędzierska and Hayes, 2016). Importantly, we recently documented the presence of a novel TA system in *Hp* encoded by *HP0968-HP0967* (Cárdenas-Mondragón et al., 2016), which was strongly expressed in bacteria forming mature biofilm on abiotic surface, but not in planktonic growth. In addition, the expression of this TA was significantly increased after contact with gastric epithelial cell line AGS. These results suggest that *HP0968-HP0967* may be expressed in the gastric mucosa and have a role in the gastric carcinogenic pathways.

## Potential Role of Polymicrobial Biofilms in Carcinogenesis

### Gastric Microbiome of *Hp*-Infected Humans

Bacterial-bacterial interactions are a major driver of pathophysiological consequences of poly-microbial biofilms, as observed in niches like oral cavity and intestine (Larsen and Fiehn, 2017; Li et al., 2017). It is not known whether this is also the case for the stomach, although there is evidence that supports the presence of such interactions in animal models. Interestingly, in *Hp*-infected INS-GAS mouse models, gastric intraepithelial neoplasia (GIN) developed much earlier in the presence of commensals, than in mice lacking commensals (Lofgren et al., 2011). A subsequent study confirmed that the presence of only a few other bacterial species had a similar impact to that of full commensals on GIN development (Lertpiriyapong et al., 2014) and that co-infection specifically promoted progression of metaplasia and foveolar hyperplasia to dysplasia (Pinzon-Guzman et al., 2018). However, to date clinical observations or *in vitro* and *in vivo* experiments for *Hp* biofilm studies have been primarily limited to be mono-microbial, exclusively to *Hp*. There are only a couple of studies that demonstrated the presence of non-*Hp* bacterial aggregates in the gastric mucus layer and crypts of the patients during acid suppression treatment or with gastric primary lymphoma, using chemical and immunochemical staining (Jonkers et al., 1997; Sanduleanu et al., 2001). These studies reported that almost two thirds of the patients harbored non-*Hp* bacteria and that co-existence of *Hp* and non-*Hp* bacteria was common (Jonkers et al., 1997; Sanduleanu et al., 2001), although the presence of EPS between bacteria was not examined.

Recent studies based on *16S* rRNA gene sequencing or other high dimensional approaches to characterize the gastric microbiome have revealed that the gastric lumen is inhabited by a wide range of commensal bacteria, contrary to the previous belief that high gastric acidity kills most microorganisms (Alarcón et al., 2017; Yu et al., 2017). Gastric microbiome comprises commensals from the ororespiratory tract through ingestion, as well as in a smaller fraction from the intestinal tract by biliary reflux (Sanduleanu et al., 2001; Yu et al., 2017). Some of these bacteria are just transient, and data pointing to which are truly resident of the gastric mucosa, other than *Hp*, are still limited. Multiple studies have found that the dominant genera in the gastric mucosa comprise *Streptococcus, Lactobacillus, Rothia, Prevotella, Veillonella, Neisseria*, and *Haemophilus*, including over 100 species (Sheh and Fox, 2013; Alarcón et al., 2017; Yu et al., 2017). There is also a consensus that *Hp* infection, especially before the development of gastric atrophy, substantially reduces diversity and richness of gastric microbiome by dominating others (Alarcón et al., 2017; Parsons et al., 2017). Yet, these studies raise the possibility that some non-*Hp* gastric bacteria may cohabit with *Hp* in biofilms and that the interactions between them may play a role in gastric carcinogenesis. Differential compositions of non-*Hp* gastric microbiome have been reported between normal individuals, pre-malignant lesions and gastric cancer, although the data have been inconsistent concerning specific bacteria associated with the stage of gastric lesions and it is still unclear whether the observed differences in microbiota are a cause or consequence of carcinogenesis (Sheh and Fox, 2013; Aviles-Jimenez et al., 2014; Dias-Jacome et al., 2016; Alarcón et al., 2017). This could suggest a progressive shift in gastric microbiota structure in carcinogenesis, possibly resulting from a complex cross-talk between gastric microbiota and *Hp*, which are likely to occur within biofilms where they live in proximity. In fact, abundance of non-*Hp* bacteria may not be important if they act like a keystone pathogen, as it is well known for *Porphyromonas gingivalis* in dental biofilms (Hajishengallis et al., 2011; Lamont and Hajishengallis, 2015). Keystone pathogens are typically present at very low abundance in a particular microbial niche; still, they can alter commensal structure to become a dysbiotic community and by attracting more pathogenic microbes (Hajishengallis et al., 2011; Lamont and Hajishengallis, 2015). Such biofilms are thought to promote host inflammatory responses and immune suppression, leading to tissue damage and development of precancerous lesions.

### Commensal-*Hp* Interactions

Potential interactions between *Hp* and other commensals or pathogens have been investigated mainly in co-culture studies and also by virtual bioinformatic models. Das et al. analyzed 16S rRNA gene pyrosequencing data from 39 Indian patients with suspected *Hp* infection using a network analyses (Das et al., 2017). Their results suggest that *Hp* has negative interactions with most members of the gastric microbiota, while other microbes interacted positively with each other, showing frequent intra-cluster co-occurrence/co-operation and increased network density. Major microbes that showed negative interactions with *Hp* include *Ralstonia, Bradyrhizobium, Cloacibacterium, Acidovorax, Aeromonas, Halomonas, Bacillus, Methylobacterium*, and *Meiothermus.* On the other hand, Krausse et al. (2005) examined the effects of other bacteria found in the gastric lumen on the growth of 30 *Hp* clinical isolates and one reference strain, using cross-streak *in vitro* culture. Among 29 bacteria tested, *Staphylococcus epidermidis, Staphylococcus aureus, Pseudomonas aeruginosa, Stenotrophomonas maltophilia, Morganella morganii, Serratia marcescens, Bf, Fn*, and *Clostridium difficile* showed the strongest growth inhibition, with varied degree on the different *Hp* strains (Krausse et al., 2005). Other researchers have focused on bacteria more common in oral cavity, *Streptococcus* and *Lactobacillus*. When a *cagA* positive *Hp* strain was co-cultured with human monocyte-derived dendritic cells (DC) in the presence of *Lactobacillus*, maturation of DC was stimulated, producing more inflammatory cytokines, compared with *Hp* alone (Wiese et al., 2015). This suggests that lactic acid producing bacteria may enhance gastric inflammatory reactions caused by *Hp* and may also promote *Hp*-induced carcinogenesis. These results were consistent with a gastric microbiome study in humans showing increased abundance of *Lactobacillus* in *Hp*-associated intestinal metaplasia and intestinal type of gastric cancer, compared with non-atrophic gastritis (Aviles-Jimenez et al., 2014) as well as the increased gastric *Lactobacillus* population in INS-GAS mouse model co-infected with *Hp* and limited commensals (*Bacteroides, Clostridium*, and *Lactobacillus*) that developed GIN (Lertpiriyapong et al., 2014). However, others have reported a probiotic *Lactobacillus* strain that inhibited the colonization of *Hp* in a Mongolian gerbil model (Merino et al., 2018). More relevant to biofilm-associated *Hp*, Khosravi et al. (2014) reported that *Streptococcus mitis* induced *Hp* conversion to coccoid cells in co-culture studies and their proteomic analysis revealed a metabolic crosstalk between these two bacteria, suggesting a probable impact on *Hp*-associated carcinogenesis (Khosravi et al., 2016; Krzyżek, 2017). Furthermore, a recent animal experiment demonstrated that INS-GAS mice co-infected with *Hp* and *Streptococcus salivarius* developed more severe inflammation, hyperplasia, and dysplasia in the stomach when compared with *Hp* only at 5 months post-infection. These studies address only a small fraction of possible interactions between *Hp* and other bacteria, but provide evidence suggesting that bacterial-bacterial interactions may modify *Hp*-associated carcinogenesis (Shen et al., 2018).

Importantly, biofilms are considered to foster horizontal gene transfer between and within species through transduction and transformation and increase fitness of organisms (Madsen et al., 2012). Mixed infection with multiple *Hp* strains has been found to produce biofilms with higher adherent capacity compared with each strain alone (Grande et al., 2012). Indeed, *Hp* exhibits high homologous recombination rates (Suerbaum et al., 1998; Falush et al., 2001) and is competent for DNA uptake (Chattopadhyay et al., 2018) as the genome often contains pathogenicity islands (cagPAI) to encode a DNA transfer apparatus, such as *tfs4*, to facilitate conjugation (Fischer et al., 2010; Fernandez-Gonzalez and Backert, 2014). Conjugative DNA transfer occurred between *Campylobacter jejuni and Hp* in an experimental system (Oyarzabal et al., 2007) and inter-species horizontal gene transfer and DNA recombinatorial events have been demonstrated within *Hp* species or with closely related genera (Eppinger et al., 2004; Saunders et al., 2005). Thus, such heightened genetic exchanges are likely to promote bacterial persistence and virulence, as evidenced by acquisition of cagPAI more than 60,000 years ago (Grande et al., 2012; Fernandez-Gonzalez and Backert, 2014).

## Concluding Remarks

As discussed above, emerging evidence suggests that certain low abundance gut bacteria, such as *Fn* and *SGG*, which are capable of causing biofilm-associated infection, may promote the development of colorectal cancer in humans. This notion is supported by not only epidemiological studies, but also biological data to corroborate molecular pathways to mediate biofilm formation and bacterial aggregation. On the other hand, despite a substantial number of studies to support the ability of *Hp* to form biofilms in *in vitro* environments (Garcia et al., 2014; Hathroubi et al., 2018a), *in vivo* observations of gastric biofilms from human subjects are still very sparse. Studies based on other anatomic niches, such as gut and oral cavity indicate that behavior of biofilm-associated bacteria is different from that of planktonic counterparts (Gabrilska and Rumbaugh, 2015; Liu et al., 2016) and that bacterial-bacterial and host-bacterial interactions taking place in the biofilm community enhance virulence and adverse host responses, probably contributing to cancer initiation and progression (Li et al., 2017). Accordingly, it is highly plausible that the presence of *Hp* biofilms predisposes individuals to progression to gastric cancer, compared to infection with planktonic *Hp*, directly through altered virulence or/and indirectly through prolonged exposure due to persistent infection. However, information available to date is insufficient to test these hypotheses and thus more clinical observations are urgently needed. New histological techniques to preserve mucus layers of gastro-intestinal mucosa may be explored to gain better resolution and quantitation of *Hp*-biofilms in such studies. Furthermore, we need to accumulate more information regarding characteristics of *Hp* biofilms including compositions and biological properties of EPS and non-*Hp* microbiome, as well as geographical distribution of biofilms in relation to gastric gland types and structures and to gastric pathologies (e.g., premalignant lesions). We need also to address the possible effects of *Hp* morphological changes on host oncogenic signaling pathways. Identification of specific *Hp* gene variants resulting into enhanced biofilm formation would be helpful not only for screening patients at high risk for sequelae from *Hp* infection, but also for development of new antibiotic regimens to avoid resistance, regardless of its association with gastric cancer. Given putative auxiliary functions of type IV secretion system pili in the contact and adhesion to host and other bacterial cells (Backert et al., 2008; Maldarelli et al., 2016) and because of well established oncogenic properties of CagA, studies focused on *cagA*-positive *Hp* in high risk populations would be particularly advantageous in addressing potential effects of *Hp* biofilms on gastric carcinogenesis.

## Ethics Statement

The original clinical studies where human samples were collected were approved by the respective institutional ethical committee in Mexico and Colombia.

## Author Contributions

IK conceptualized the manuscript. JT and MC-P carried out the experiments presented in Figure 1–4. FC, JT, MB, and CR contributed to *Hp* sequence analyses. IK, EK, JT, MC-P, and CR conducted literature review and prepared the first draft. FC and MB participated in editing the draft. All authors approved the final manuscript for submission.

## Conflict of Interest Statement

The authors declare that the research was conducted in the absence of any commercial or financial relationships that could be construed as a potential conflict of interest.
